# Supplementary material for: Natural variation in BnaA07.MKK9 confers resistance to Sclerotinia stem rot in oilseed rape
Source: Nat Commun. 2024 Jun 13;15:5059. doi: 10.1038/s41467-024-49504-6 (PMC11176195; doi:10.1038/s41467-024-49504-6)
Supplement: Supplementary file 1 — Supplementary Information [file 41467_2024_49504_MOESM1_ESM.pdf]

Supplementary information

a

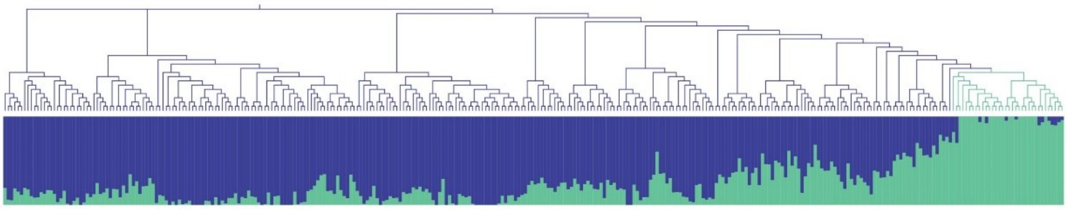

b

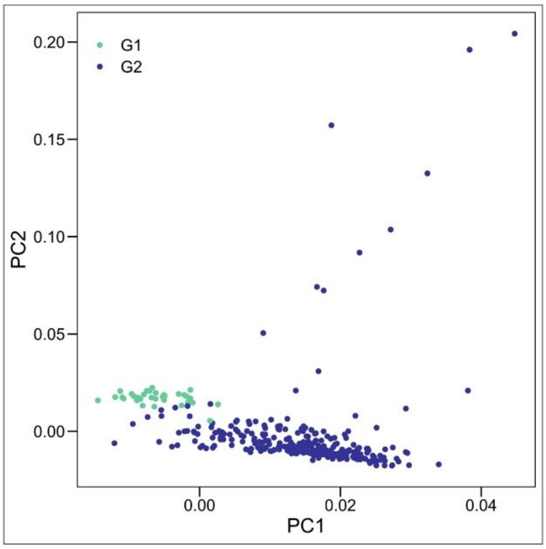

Supplementary Fig. 1 Population structure of 322 *B. napus* accessions. (a) Phylogenetic tree and structure analysis of 322 accessions. Structure analysis were performed using ADMIXTURE with K = 2. Each bar represents an accession, and the different colors correspond to its proportion of different groups. (b) Principal component analysis plot of all the accessions. Source data are provided as a Source Data file.

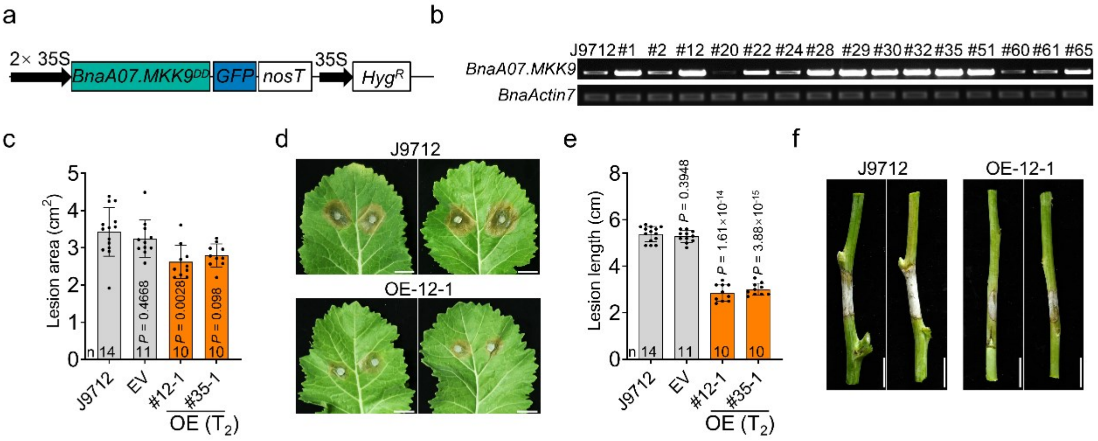

Supplementary Fig. 2 Overexpression of *$BnaA07.MKK9^{DD}$* in oilseed rape increased resistance to *S. sclerotiorum*. (a) Schematic of the *$BnaA07.MKK9^{DD}$*-Overexpression (OE) construct. 35S: cauliflower mosaic virus 35S promoter; *GFP*: green fluorescent protein; *NosT*: nopaline synthase gene terminator; and *Hyg^R*: *hygromycin B* resistance gene. (b) Expression level of *BnaA07.MKK9* in *$BnaA07.MKK9^{DD}$*-OE transgenic plants (T_0 progeny) by RT-PCR. *BnaActin7* was the reference gene. (c-f) Evaluation of Sclerotinia stem rot (SSR) resistance of the *BnaA07.MKK9* -OE lines (T_2 progenies) on leaves at the seedling stage (c, d) and on stems at the mature stage (e, f). Disease lesion on leaves and stems were photographed and quantified at 48 h post-inoculation (hpi) and 168 hpi, respectively. Empty vector (EV) transgenic plants and non-transgenic line J9712 were used as controls, scale bars: 1 cm (d) and 5 cm (f). In (c and e), each bar represents the mean ± SD, and *n* represents the number of plants, which is labeled on each bar. Statistical significance was determined by a two-tailed Student's *t*-test. Significance compared with J9712. Source data are provided as a Source Data file.

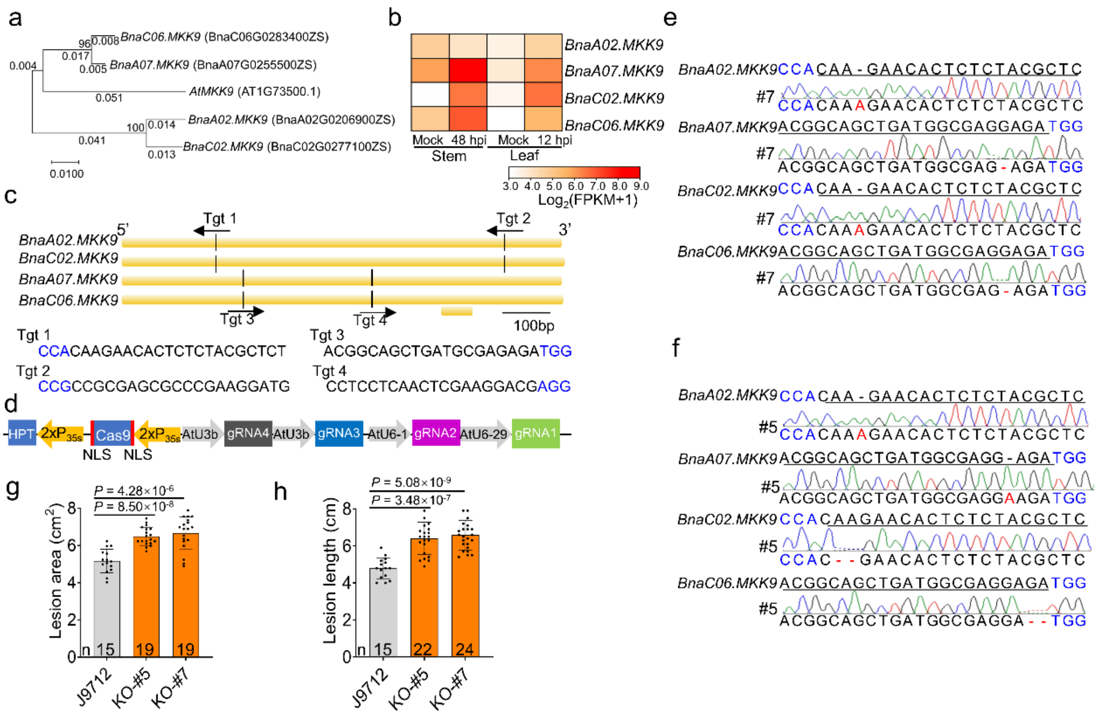

Supplementary Fig. 3 Generation of *Bnamkk9* mutants by CRISPR/Cas9 gene editing. (a) A phylogenetic tree was constructed based on a protein sequence alignment of the four *BnaMKK9* homologs using the neighbor-joining method with 1,000 bootstrap replicates in MEGA7. Values at the nodes represent the percent bootstrap confidence level. (b) Expression patterns of *BnaMKK9* in response to *S. sclerotiorum* infection using RNA-seq data. FPKM, fragments per kilobase of transcript per million fragments mapped. (c) Schematic of the *BnaMKK9* gene model and the four target sites (Tgt1-4). Exons are shown as yellow boxes. Vertical lines indicate target sites, the arrows indicate the sgRNA direction, and the protospacer-adjacent motif (PAM) is marked in blue. Scale bars: 100 bp. (d) Schematic of the CRISPR/Cas9 construct. NLS: nuclear location signal. P_{35S}: cauliflower mosaic virus 35S promoter. AtU3: *Arabidopsis* U3 promoter. AtU6: *Arabidopsis* U6 promoter. HPT: resistance to hygromycin B for transgenic plant selection. (e, f) Sanger sequencing chromatograms of the two *Bnamkk9* progenies (T_4). Target sequences are underlined, nucleotide indels are red, and PAMs are blue. (g, h) Evaluation of Sclerotinia stem rot (SSR) resistance of the *Bnamkk9* mutants on leaves at 48 h post-inoculation (hpi) (g) and on stems at 168 hpi (h) in T_5 progenies in 2021. Each bar represents the mean ± SD, and *n* represents the number of plants, which is labeled on each bar. Statistical significance was determined by a two-tailed Student's *t*-test. Significance compared with J9712. Source data are provided as a Source Data file.

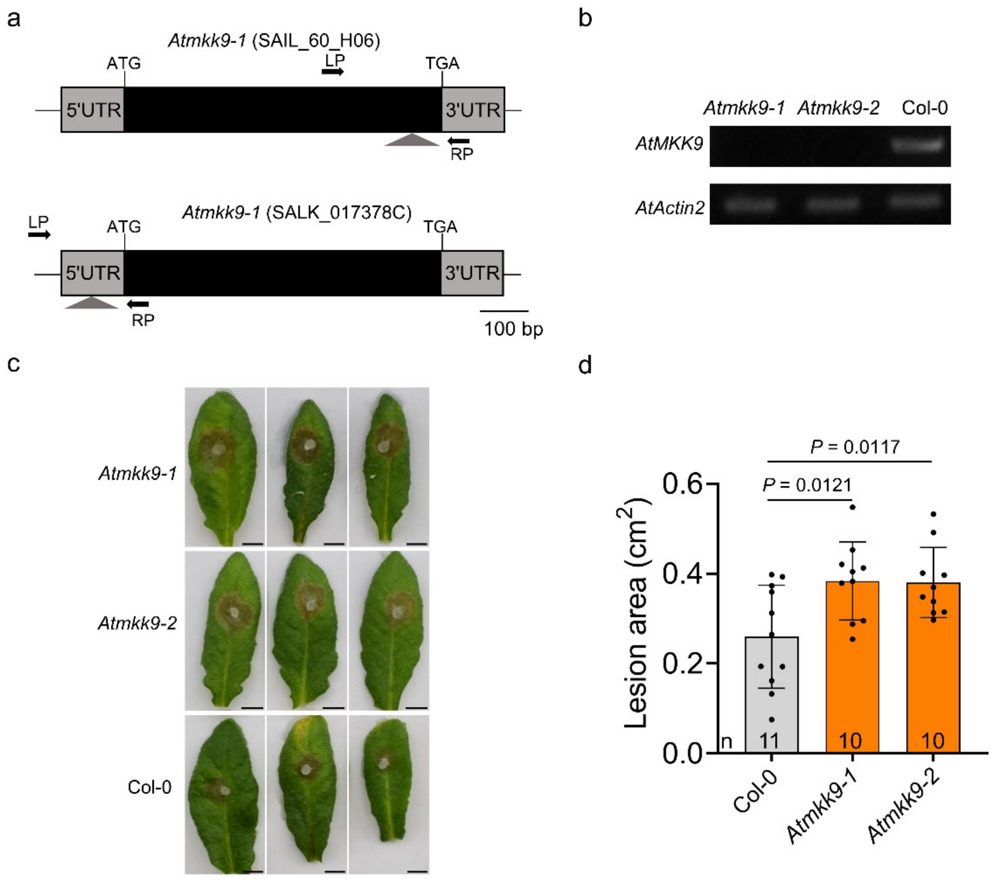

Supplementary Fig. 4 Sclerotinia stem rot (SSR) resistance of the two *Atmkk9* mutants in *Arabidopsis*. (a) Schematic of *AtMKK9* and the position of the T-DNA insertion for the *Atmkk9-1* and *Atmkk9-2* mutants. Black and gray boxes indicate exons and 5'- and 3'- untranslated regions (UTRs), respectively. T-DNA insertions and locations of the left primers (LP) and right primers (RP) used for genotyping are shown as triangles and arrows, respectively. Scale bar: 100 bp. (b) Expression levels of *AtMKK9* in Col-0, *Atmkk9-1*, and *Atmkk9-2* by RT-PCR. *AtActin2* was the internal control. (c, d) Evaluation of SSR resistance in leaves of Col-0, *Atmkk9-1*, and *Atmkk9-2*. Disease lesions on leaves were photographed (c) and quantified (d) at 30 h post-inoculation (hpi). Scale bars in (c): 1 cm. In (d), bars represent the mean ± SD, and *n* represents the number of plants, which is labeled on each bar. Statistical significance was determined by a two-tailed Student's *t*-test. Significance compared with Col-0. Source data are provided as a Source Data file.

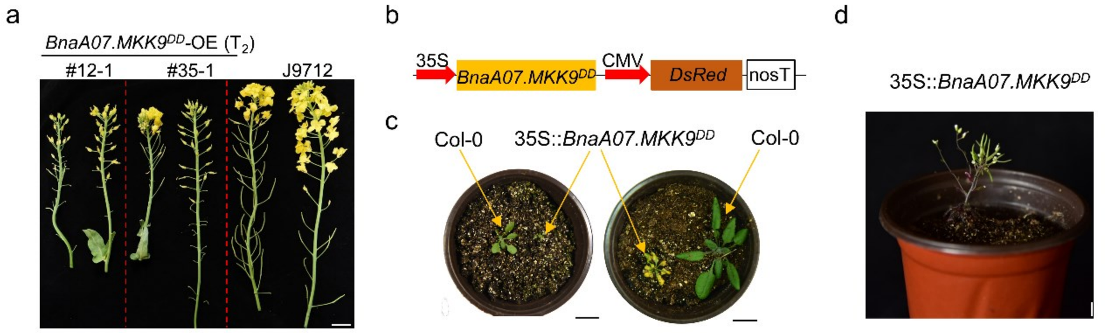

Supplementary Fig. 5 Growth phenotype of *BnaA07.MKK9^{DD}*-overexpression (OE) transgenic plants in oilseed rape and *Arabidopsis*. (a) Growth phenotypes of *BnaA07.MKK9^{DD}*-OE transgenic plants (T_2 progenies) and J9712 at the flowering stage. Scale bars: 5 cm. (b) Schematic of the *BnaA07.MKK9^{DD}*-OE construct in *Arabidopsis*. CMV: cytomegalovirus promoter, *DsRed*: DsRed marker for transgenic plant selection. 35S: cauliflower mosaic virus 35S promoter. (c-d) Growth phenotypes of *Arabidopsis* 35S::*BnaA07.MKK9^{DD}* transgenic plants and Col-0 at seedling and flowering stages. Scale bars: 1 cm.

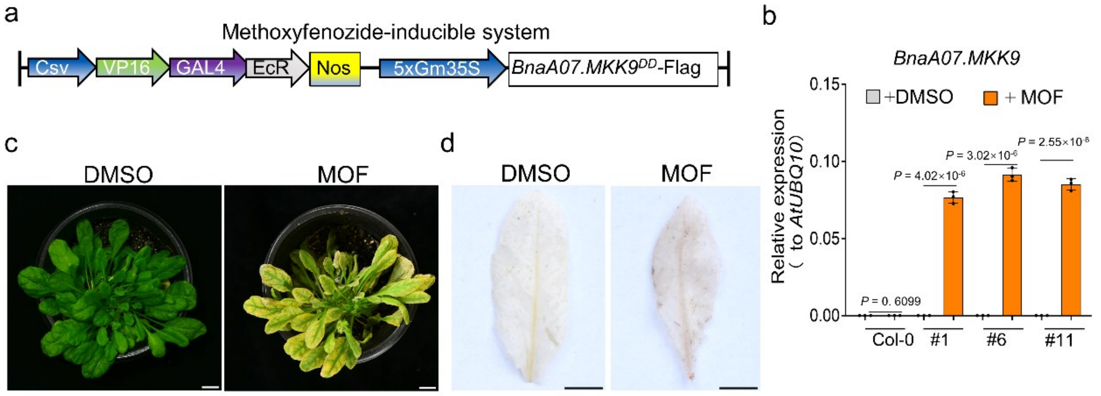

Supplementary Fig. 6 Schematic of the methoxyfenozide (MOF)-inducible expression system construct and the growth phenotypes of *Csv::BnaA07.MKK9^{DD}* *Arabidopsis* plants. (a) Schematic of *Csv::BnaA07.MKK9^{DD}*. Csv: cassava vein mosaic virus promoter. VP16: protein expression activation domain. GAL4: DNA binding-domain in yeast. EcR: Ecdysone receptor. 5 × GmV35S: five copies of the 102-bp GAL4 response elements fused to the CaMV35S minimal promoter. (b) Relative expression of *Csv::BnaA07.MKK9^{DD}* transgenic *Arabidopsis* lines (#1, #6, and #11) and Col-0 treated with either DMSO (control) or 61.3 μM methoxyfenozide (MOF). Samples were taken 8 h post-treatment, with *AtUBQ10* serving as the reference gene for expression analysis. Values are means ± SD (n = 3 biological replicates). (c, d) Phenotypes (c) and 3, 3'-diaminobenzidine (DAB) staining (d) of *Csv::BnaA07.MKK9* plants at 72 h after MOF treatment. Scale bars: 1 cm. Source data are provided as a Source Data file.

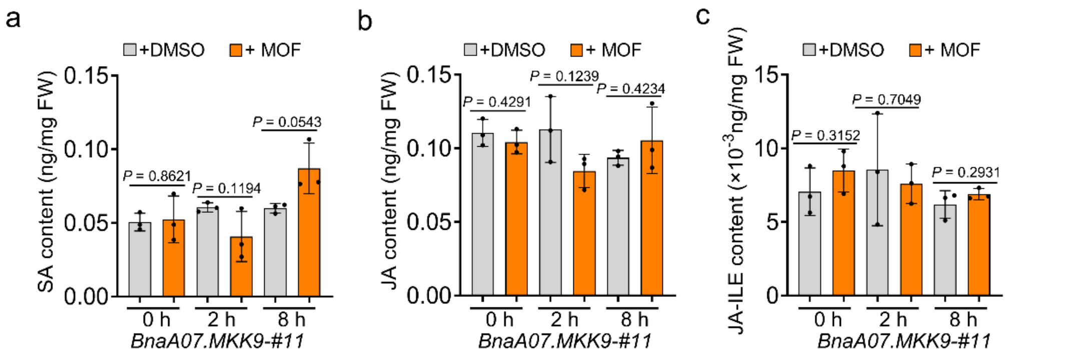

Supplementary Fig. 7 Plant hormone contents in the leaves of *Csv::BnaA07.MKK9^{DD} Arabidopsis* plants. (a-c) Salicylic acid (SA, a), jasmonic acid (JA, b) and jasmonoyl isoleucine (JA-ILE, c) contents in *Csv::BnaA07.MKK9^{DD}* plants at 0, 2 and 8 h after DMSO (control) or methoxyfenozide (MOF) treatment. Each bar represents the mean ± SD (*n* = 3 biological replicates). Statistical significance was determined by a two-tailed Student's *t*-test; FW: fresh weight. Source data are provided as a Source Data file.

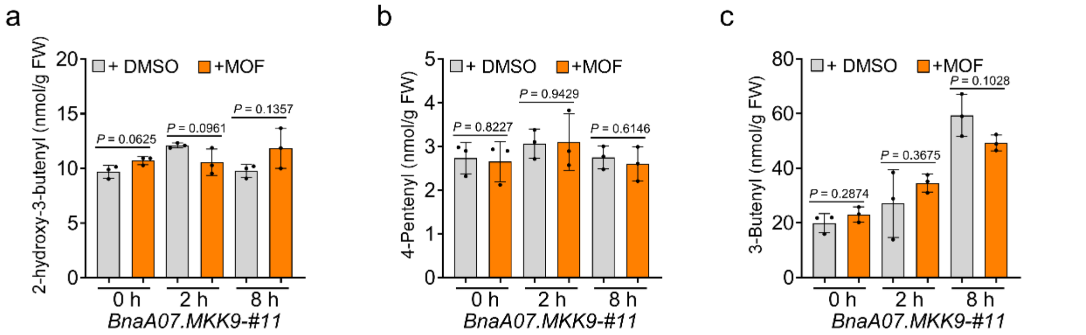

Supplementary Fig. 8 Aliphatic glucosinolates (AGSs) contents in *Csv::BnaA07.MKK9^{DD} Arabidopsis* plants. 2-Hydroxy-3-butenyl (a), 4-Pentenyl (b), and 3-Butenyl (c) contents in *Csv::BnaA07.MKK9^{DD}* plants at 0, 2 and 8 h after DMSO (control) or methoxyfenozide (MOF) treatment. Each bar represents the mean ± SD (*n* = 3 biological replicates). Statistical significance was determined by a two-tailed Student's *t*-test; FW: fresh weight. Source data are provided as a Source Data file.

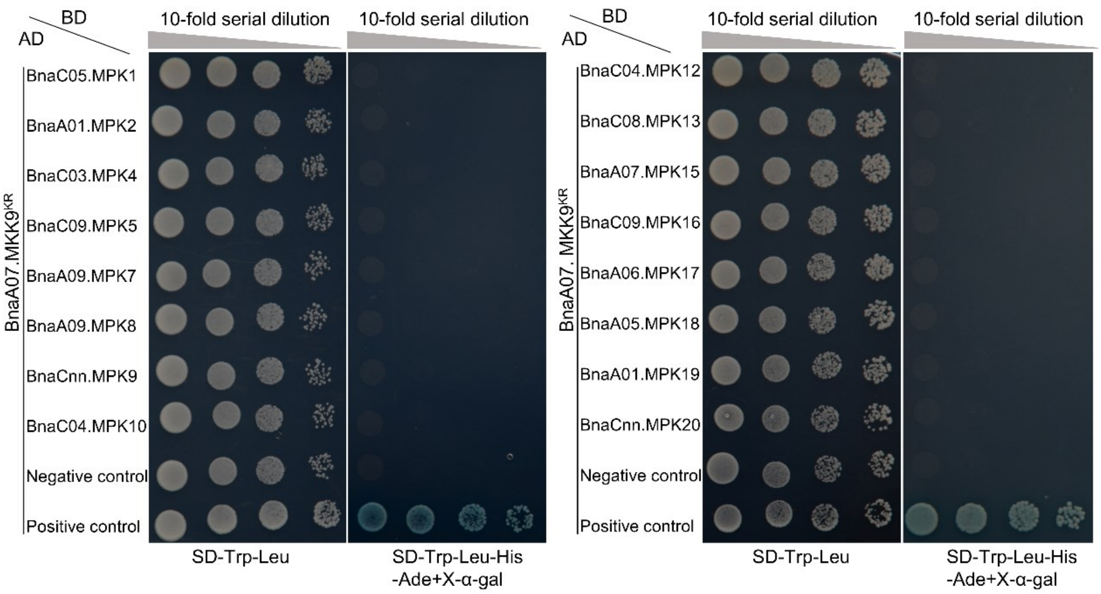

Supplementary Fig. 9 Yeast two-hybrid (Y2H) assays showing the interactions between BnaA07.MKK9^{KR} and BnaMAPKs. Yeast cells were grown on synthetic defined (SD)-Leu-Trp or SD-Leu-Trp-His-Ade/X-α-Gal medium. BD, DNA-binding domain; AD, activation domain. BD-53/AD-RecT was the positive control. BD-Lam/AD-RecT was the negative control. Experiments were repeated three times with similar results.

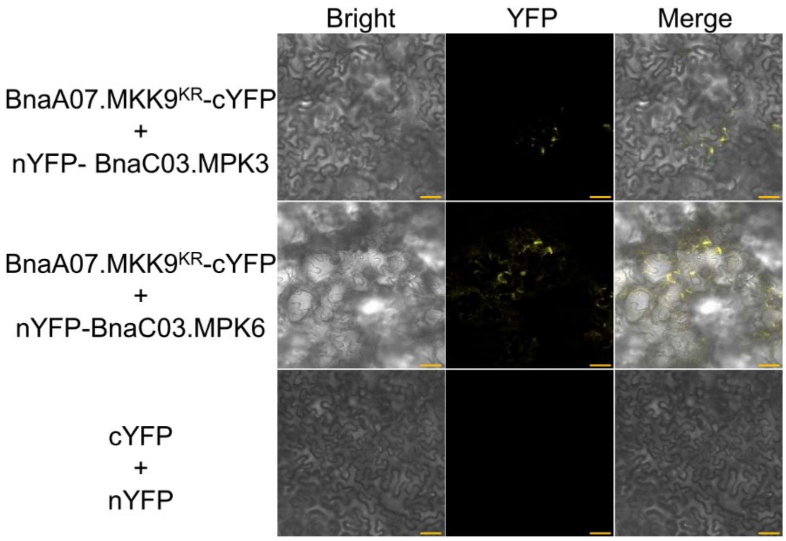

Supplementary Fig. 10 Bimolecular fluorescence complementation (BiFC) assays showing the interactions between BnaC03.MPK3/BnaC03.MPK6 and BnaA07.MKK9^{KR} in *N. benthamiana* cells. Yellow fluorescent protein (YFP) signals were detected on a confocal laser-scanning microscope. Scale bars: 50 µm. Experiments were repeated three times with similar results.

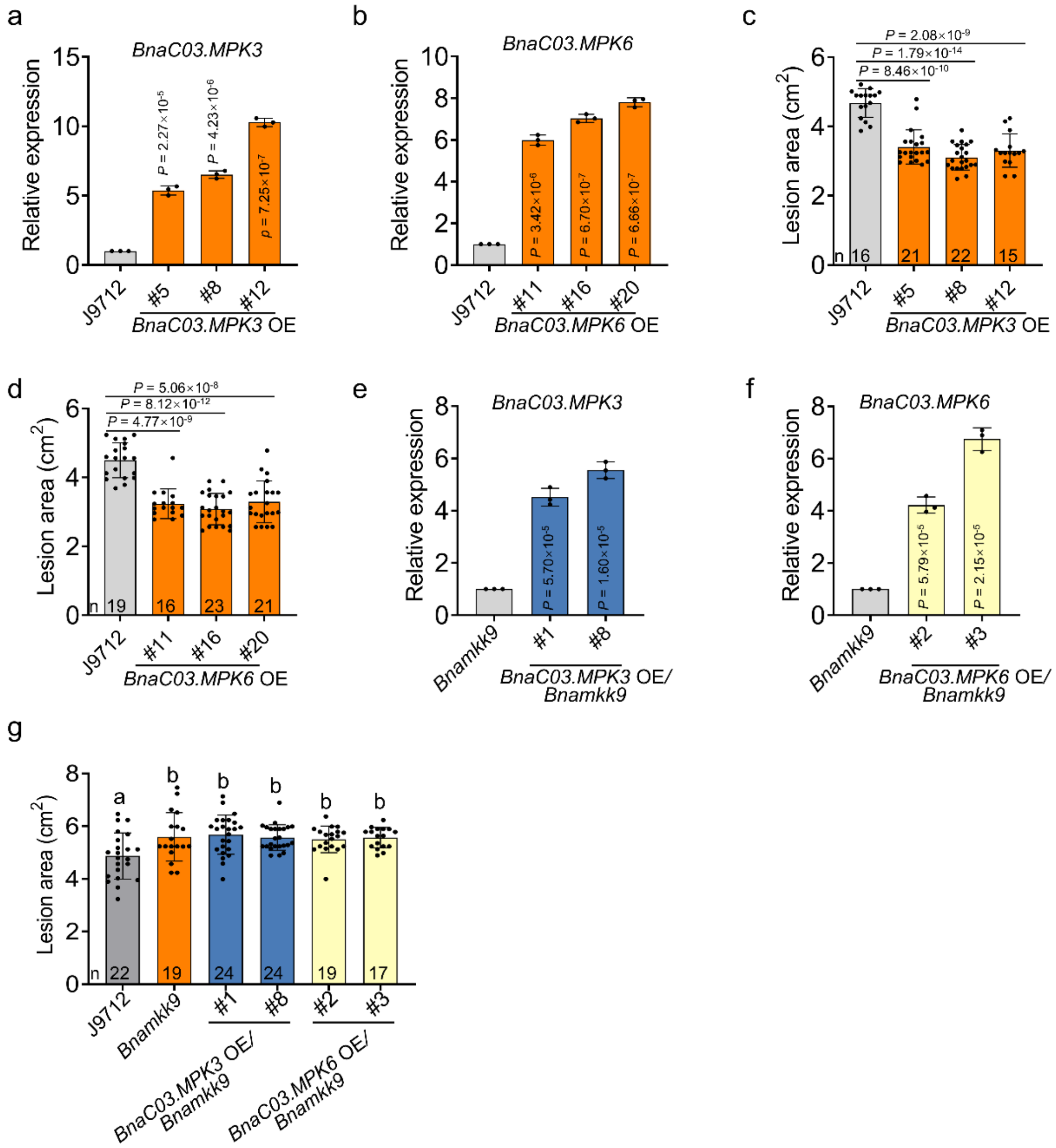

Supplementary Fig. 11 Evaluation of Sclerotinia stem rot (SSR) resistance in the *BnaC03.MPK6* OE, *BnaC03.MPK6* OE, *BnaC03.MPK6* OE/*Bnamkk9*, and *BnaC03.MPK6* OE/*Bnamkk9* plants in oilseed rape. (a-d) Relative expression (a, b) lesion areas (c, d) of *BnaC03.MPK3*/*BnaC03.MPK6* OE transgenic plants and J9712. The inoculated leaves of J9712, *BnaC03.MPK3* OE (T_2 progenies, c) and *BnaC03.MPK6* OE (T_2 progenies, d) plants were photographed and quantified at 48 hours post-inoculation (hpi). Each bar represents the mean ± SD (n = 3 biological replicates in a and b). (e-g) Relative expression (e, f) and lesion areas (g) of J9712, *Bnamkk9*, *BnaC03.MPK3* OE/*Bnamkk9*, and *BnaC03.MPK6* OE/*Bnamkk9* (T_2 progenies). In (e, f), each bar represents the mean ± SD (n = 3 biological replicates). In (c, d, g), the *S. sclerotiorum* inoculated leaves were photographed and quantified at 48 hpi. Each bar represents the mean ± SD, and n represents the number of plants, which is labeled on each bar. For qRT-PCR in (a, b, e, f), *BnaUBC10* was the reference gene. In (a-f), statistical significance was determined by a two-tailed Student's t-test. In (a-d), significance compared with J9712. In (e, f), significance compared with *Bnamkk9*. In

(g), different lowercase letters indicate significant differences (P < 0.05), determined with one-way analysis of variance (ANOVA) with Tukey's honestly significant difference (HSD) test. Source data are provided as a Source Data file.

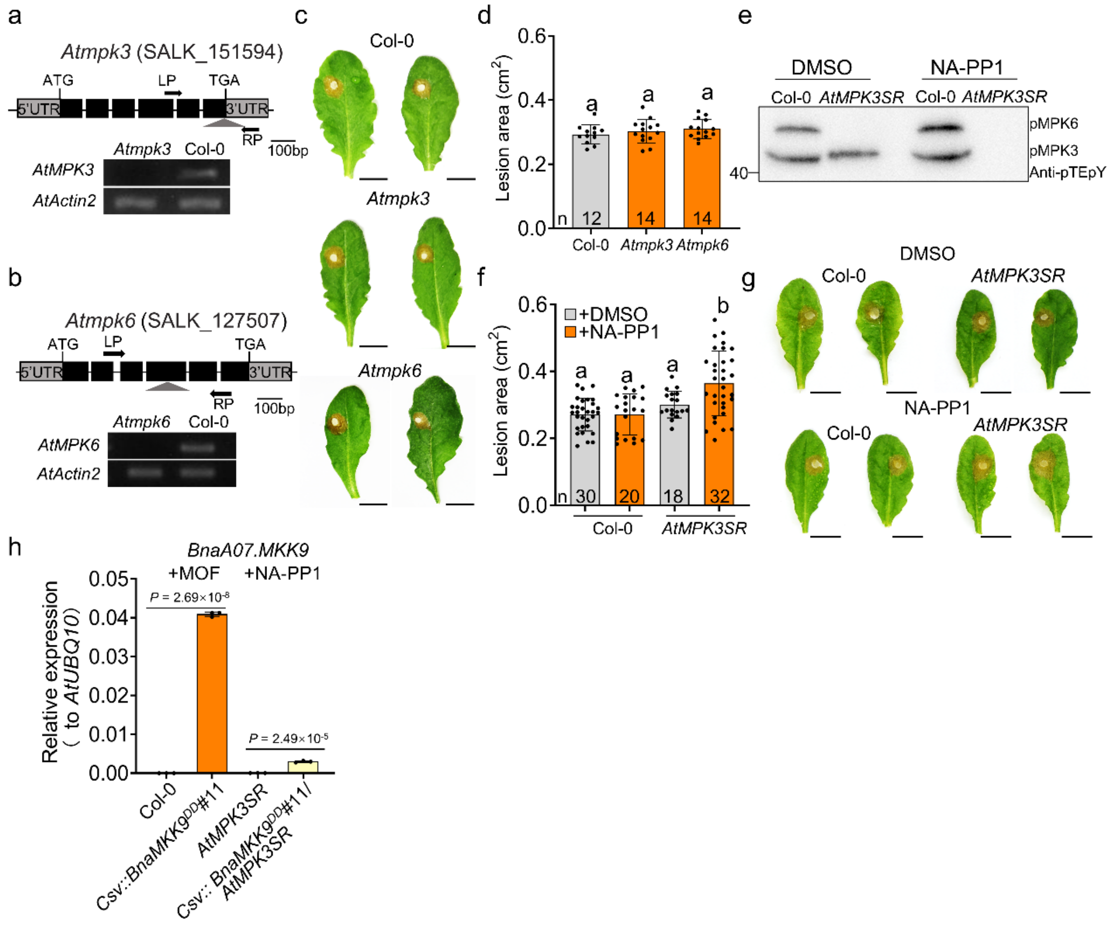

Supplementary Fig. 12 *AtMPK3* and *AtMPK6* have redundant functions in Sclerotinia stem rot (SSR) resistance. (a-b) Schematics of the T-DNA insertions for *Atmpk3* (a) and *Atmpk6* (b) mutants. Exons and 5'- and 3'- untranslated regions (UTRs) are shown as black and gray boxes, respectively. The T-DNA insertions and locations of the the left primers (LP) and right primers (RP) used for genotyping are shown as triangles and arrows, respectively. Scale bar: 100 bp. Expression levels of *AtMPK3* and *AtMPK6* in Col-0, *Atmpk3* and *Atmpk6* mutants by RT-PCR, with *AtActin2* as the internal control. (c-d) Disease symptoms of Col-0, *Atmpk3* and *Atmpk6* mutants. Disease lesions were photographed (c) and quantified at 30 hours post-inoculation (hpi, d). Scale bars: 1 cm. (e) Kinase activity of MPK3 and MPK6 in Col-0 and *AtMPK3SR* at 12 h after *S. sclerotiorum* inoculation. Col-0 and *AtMPK3SR* leaves were pretreated with DMSO or NA-PP1 for 12 h before *S. sclerotiorum* inoculation. MPK3 and MPK6 activity were determined by immunoblot analysis using anti-pTEpY antibody. Molecular mass markers in kilodaltons are shown on the left. (f-g) Evaluation of SSR resistance of Col-0 and *AtMPK3SR* mutants in leaves. Disease lesions were

photographed (g) and quantified (f) at 30 hpi. Scale bars: 1 cm. *S. sclerotiorum* inoculations were performed at 12 h after DMSO or NA-PP1 treatment. (h) Relative expression of *BnaA07.MKK9* in Col-0, *Csv::BnaA07.MKK9*^{DD}, *AtMPK3SR* and *Csv::BnaA07.MKK9*^{DD}*/AtMPK3SR Arabidopsis* plants. qRT-PCR was performed at 12 h after methoxyfenozide (MOF) and NA-PP1 treatment before *S. sclerotiorum* inoculation. *AtUBQ10* served as the reference gene. Each bar represents the mean ± SD (n = 3 biological replicates). Statistical significance was determined by a two-tailed Student's t-test. In (d, f), each bar represents the mean ± SD, and n represents the number of plants, which is labeled on each bar. Different lowercase letters indicate significant differences (P < 0.05), determined with one-way analysis of variance (ANOVA), Tukey's honestly significant difference (HSD) test. Source data are provided as a Source Data file.

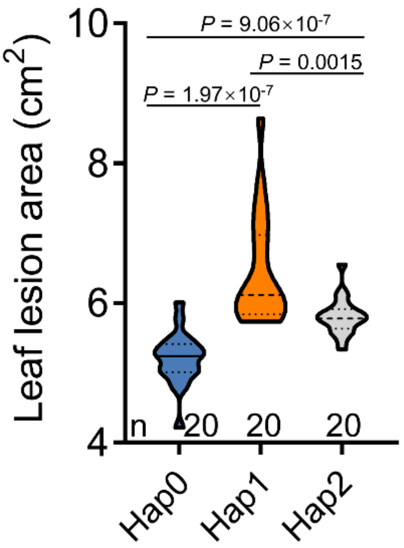

Supplementary Fig. 13 Sclerotinia stem rot (SSR) resistance of 20 randomly selected accessions for each haplotype evaluated by detached-leaf inoculations. The violin plots in show the median (the solid line in the middle) and the lower and upper quartiles (the dashed lines). The shape of the violin plots represents data distribution, and the bounds indicate the minima and maxima (n = 20 accessions.). Statistical significance was determined by a two-tailed Student's t-test. Source data are provided as a Source Data file.

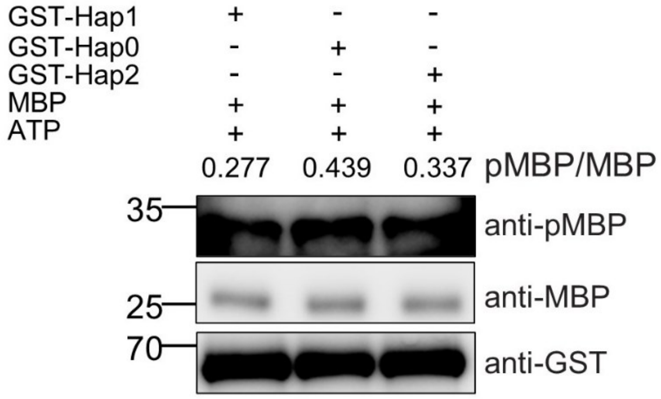

Supplementary Fig. 14 Kinase activity of the three BnaA07.MKK9 haplotypes *in vitro*. Purified GST-Hap0, GST-Hap1, and GST-Hap2 proteins were used and myelin basic protein (MBP) was the substrate. Anti-pMBP was used to detect the phosphorylation of MBP. Loading controls were detected using anti-GST and anti-MBP, respectively. Values indicate the relative band density of phosphorylated MBP normalized to MBP protein levels using ImageJ software. Molecular mass markers in kilodaltons are shown on the left. Experiments were repeated three times with similar results. Source data are provided as a Source Data file.

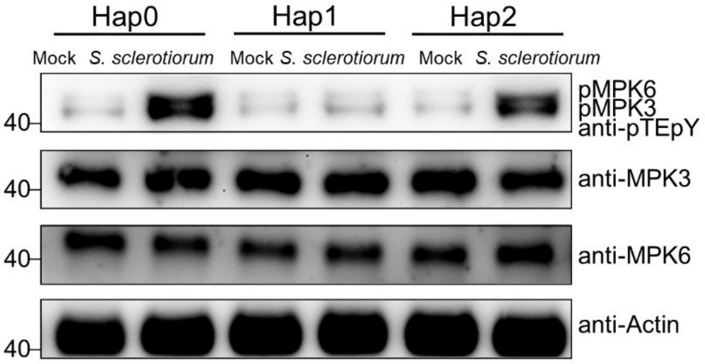

Supplementary Fig. 15 BnaMAPK3/6 activity in the three *BnaA07.MKK9* haplotypes at 12 hpi with *S. sclerotiorum* or the mock inoculation. Phosphorylation of BnaMPK3/6 was detected by immunoblotting (IB) with the anti-pTEpY antibody and protein levels detected with anti-BnaMPK6 and anti-BnaMPK3 antibodies. Actin served as the loading control. Molecular mass markers in kilodaltons are shown on the left. Experiments were repeated three times with similar results. Source data are provided as a Source Data file.
