# Supplementary material for: Natural variation in BnaA07.MKK9 confers resistance to Sclerotinia stem rot in oilseed rape
Source: Nat Commun. 2024 Jun 13;15:5059. doi: 10.1038/s41467-024-49504-6 (PMC11176195; doi:10.1038/s41467-024-49504-6)
Supplement: Supplementary file 3 — Description of Additional Supplementary Files [file 41467_2024_49504_MOESM3_ESM.pdf]

<u>Description of Additional Supplementary Files</u>

Supplementary Data Legends

Supplementary Data 1: List of 322 accessions for genome-wide association studies in this study.

Supplementary Data 2: Genotyping of BnaMKK9 mutants and their transmission to T1, T2, and T4 generations.

Supplementary Data 3: Detection of potential offtarget sites at esch sgRNA target site of Bnamkk9 quadruple mutants (#5 and #7) in T4 progenies.

Supplementary Data 4: Agronomic traits of the J9712 and Bnamkk9 mutants in the experimental field.

Supplementary Data 5: All primers used in this study.
