# Supplementary material for: Natural variation in BnaA07.MKK9 confers resistance to Sclerotinia stem rot in oilseed rape
Source: Nat Commun. 2024 Jun 13;15:5059. doi: 10.1038/s41467-024-49504-6 (PMC11176195; doi:10.1038/s41467-024-49504-6)
Supplement: Supplementary file 9 — Reporting Summary [file 41467_2024_49504_MOESM9_ESM.pdf]

nature portfolio

Corresponding author(s): NCOMMS-23-55588

Last updated by author(s): May 16, 2024

Reporting Summary

Nature Portfolio wishes to improve the reproducibility of the work that we publish. This form provides structure for consistency and transparency in reporting. For further information on Nature Portfolio policies, see our Editorial Policies and the Editorial Policy Checklist.

Statistics

For all statistical analyses, confirm that the following items are present in the figure legend, table legend, main text, or Methods section.

n/a	Confirmed	
☐	☒	The exact sample size (*n*) for each experimental group/condition, given as a discrete number and unit of measurement
☐	☒	A statement on whether measurements were taken from distinct samples or whether the same sample was measured repeatedly
☐	☒	The statistical test(s) used AND whether they are one- or two-sided<br>*Only common tests should be described solely by name; describe more complex techniques in the Methods section.*
☒	☐	A description of all covariates tested
☐	☒	A description of any assumptions or corrections, such as tests of normality and adjustment for multiple comparisons
☐	☒	A full description of the statistical parameters including central tendency (e.g. means) or other basic estimates (e.g. regression coefficient) AND variation (e.g. standard deviation) or associated estimates of uncertainty (e.g. confidence intervals)
☐	☒	For null hypothesis testing, the test statistic (e.g. *F*, *t*, *r*) with confidence intervals, effect sizes, degrees of freedom and *P* value noted<br>*Give P values as exact values whenever suitable.*
☒	☐	For Bayesian analysis, information on the choice of priors and Markov chain Monte Carlo settings
☒	☐	For hierarchical and complex designs, identification of the appropriate level for tests and full reporting of outcomes
☒	☐	Estimates of effect sizes (e.g. Cohen's *d*, Pearson's *r*), indicating how they were calculated

Our web collection on statistics for biologists contains articles on many of the points above.

Software and code

Policy information about availability of computer code

Data collection	No software was used in data collection.
Data analysis	The following open-source software were used in data analysis:<br>BnIR database: (https://yanglab.hzau.edu.cn/)<br>CMplot package (v.4.0): (https://github.com/YinLiLin/R-CMplot/blob/master/CMplot.r)<br>PLINK (v.1.90b4.4): (https://zzz.bwh.harvard.edu/plink/data.shtml)<br>ClustalW: (https://www.genome.jp/tools-bin/clustalw)<br>R (v.x64 4.1.1): (https://www.r-project.org/)<br>TB tools: (https://github.com/CJ-Chen/TBtools-II/releases)<br>GEMMA (v0.98.1): (https://github.com/genetics-statistics/GEMMA/releases)<br>MEGA software (v7.0.21): (https://www.megasoftware.net/)<br>ADMIXTURE software(v.1.3.0) :(http://www.genetics.ucla.edu/software)<br>GCTA (v.1.94.0) software:(https://yanglab.westlake.edu.cn/software/gcta/#Overview)<br>ggplot2 packages in R (v 4.3.2) (http://had.co.nz/ggplot2/)

For manuscripts utilizing custom algorithms or software that are central to the research but not yet described in published literature, software must be made available to editors and reviewers. We strongly encourage code deposition in a community repository (e.g. GitHub). See the Nature Portfolio guidelines for submitting code & software for further information.

Data

Policy information about availability of data

All manuscripts must include a data availability statement. This statement should provide the following information, where applicable:

- Accession codes, unique identifiers, or web links for publicly available datasets
- A description of any restrictions on data availability
- For clinical datasets or third party data, please ensure that the statement adheres to our policy

The RNA-seq data generated in this study have been deposited in the National Center for Biotechnology Information (NCBI) Sequence Read Archive database under accession PRJNA1040954, PRJNA1041073, PRJNA1041558. The RNA-seq data of rapeseed stem at 48 hours post-inoculation was acquired from the NCBI Sequence Read Archive database under accession SRP053361. The raw resequencing data of 418 rapeseed accessions from different breeding periods was acquired from the NCBI Sequence Read Archive database under accessions PRJNA416679.

Research involving human participants, their data, or biological material

Policy information about studies with human participants or human data. See also policy information about sex, gender (identity/presentation), and sexual orientation and race, ethnicity and racism.

Reporting on sex and gender	n/a
Reporting on race, ethnicity, or other socially relevant groupings	n/a
Population characteristics	n/a
Recruitment	n/a
Ethics oversight	n/a

Note that full information on the approval of the study protocol must also be provided in the manuscript.

Field-specific reporting

Please select the one below that is the best fit for your research. If you are not sure, read the appropriate sections before making your selection.

☒ Life sciences ☐ Behavioural & social sciences ☐ Ecological, evolutionary & environmental sciences

For a reference copy of the document with all sections, see nature.com/documents/nr-reporting-summary-flat.pdf

Life sciences study design

All studies must disclose on these points even when the disclosure is negative.

Sample size	We used 322 rapeseed accessions for GWAS analysis. Other required experimental sample sizes were determined based on our previous studies (Wu et al., 2022, Lin et al., 2022).<br>Wu J, Yin SL, Lin L, et al. Host-induced gene silencing of multiple pathogenic factors of Sclerotinia sclerotiorum confers resistance to Sclerotinia rot in Brassica napus. Crop J, 2022, 10(3): 661-671.<br>Lin L, Fan JL, Li PP, et al. The Sclerotinia sclerotiorum-inducible promoter pBnGH17D7 in Brassica napus: isolation, characterization, and application in host-induced gene silencing. J Exp Bot, 2022, 73(19): 6663-6677.
Data exclusions	No data was excluded from our analysis.
Replication	All experiments were repeated 3-4 times, and we have stated the number of replications in the corresponding figure legends and methods section.
Randomization	For in-field studies, all rapeseed plants were planted in randomized blocks.
Blinding	Data collection and analysis were performed in a blinded manner.

Reporting for specific materials, systems and methods

We require information from authors about some types of materials, experimental systems and methods used in many studies. Here, indicate whether each material, system or method listed is relevant to your study. If you are not sure if a list item applies to your research, read the appropriate section before selecting a response.

Materials & experimental systems

n/a	Involved in the study
☐	☒ Antibodies
☒	☐ Eukaryotic cell lines
☒	☐ Palaeontology and archaeology
☒	☐ Animals and other organisms
☒	☐ Clinical data
☒	☐ Dual use research of concern
☐	☒ Plants

Methods

n/a	Involved in the study
☒	☐ ChIP-seq
☒	☐ Flow cytometry
☒	☐ MRI-based neuroimaging

Antibodies

Antibodies used

The commercialized antibodies were ordered from the Cowin Bio, MBL, TransGen Biotech, Cell Signaling Technology, Millipore and Sigma Aldrich. The catalog number and the specific information are listed below:anti-GST antibody (Cowin Bio, CW0084, 1:1,000 dilution), anti-His antibody (Cowin Bio, CW0285, 1:1,000 dilution), anti-Myc antibody (MBL,M192-3S, 1:1,000 dilution), anti-DDDDK tag antibody (MBL, M185- 3L, 1:1,000 dilution), anti-β-Actin antibody (TransGen Biotech, HC201-01, 1:1,000 dilution), anti-pTEpY antibody (Cell Signaling Technology, 9101S, 1:1,000 dilution), anti-pMBP antibody (Millipore, 05-429, 1:1,000 dilution), and the anti-MBP antibody (Sigma Aldrich, M3821,1:1,000 dilution).
The anti-BnaMPK3, anti-BnaMPK6 and anti-BnaMKK9 antibodies used in this study were provided by Kaijing Biotech (Shanghai, China).

Validation

The specificities of the commercialized antibodies can be found in the manufacturers' websites, listed as follows:
anti-GST antibody (Cowin Bio, CW0084): https://www.cwbio.com/goods/index/id/10104.
anti-His antibody (Cowin Bio, CW0285): https://www.cwbio.com/goods/index/id/10177.
anti-Myc antibody (MBL, M192-3S): https://www.mblbio.com/bio/g/dtl/A/?pcd=M192-3.
anti-DDDDK tag antibody (MBL, M185-3L): https://www.mblbio.com/bio/g/dtl/A/index.html?pcd=M185-3L.
anti-β-Actin antibody (TransGen Biotech, HC201-01): https://www.transgen.com/antibody_reference/393.html.
anti-pTEpY antibody (Cell Signaling Technology, 9101S): https://www.cellsignal.cn/products/primary-antibodies/phospho-p44-42-mapk-erk1-2-thr202-tyr204-antibody/9101.
anti-pMBP antibody: (Millipore, 05-429): https://www.sigmaaldrich.cn/CN/zh/product/mm/05429.
anti-MBP antibody (Sigma Aldrich, M3821): https://www.sigmaaldrich.cn/CN/zh/product/sigma/m3821.
anti-BnaMPK3 antibody (Kaijing Biotech): for detecting endogenous BnaMPK3 in rapeseed as shown in Fig. 4i and supplementary Fig. 13.
anti-BnaMPK6 antibody (Kaijing Biotech): for detecting endogenous BnaMPK6 in rapeseed as shown in Fig. 4i and supplementary Fig. 13.
anti-BnaA07.MKK9 antibody (Kaijing Biotech): for detecting endogenous anti-BnaA07.MKK9 in rapeseed as shown in Fig. 1f, g and Fig. 6d.

Dual use research of concern

Policy information about dual use research of concern

Hazards

Could the accidental, deliberate or reckless misuse of agents or technologies generated in the work, or the application of information presented in the manuscript, pose a threat to:

No	Yes
☒	☐ Public health
☒	☐ National security
☒	☐ Crops and/or livestock
☒	☐ Ecosystems
☒	☐ Any other significant area

Experiments of concern

Does the work involve any of these experiments of concern:

No	Yes	
☒	☐	Demonstrate how to render a vaccine ineffective
☒	☐	Confer resistance to therapeutically useful antibiotics or antiviral agents
☒	☐	Enhance the virulence of a pathogen or render a nonpathogen virulent
☒	☐	Increase transmissibility of a pathogen
☒	☐	Alter the host range of a pathogen
☒	☐	Enable evasion of diagnostic/detection modalities
☒	☐	Enable the weaponization of a biological agent or toxin
☒	☐	Any other potentially harmful combination of experiments and agents

Plants

Seed stocks	Arabidopsis T-DNA insertion mutant which from prior publication were listed the manuscript and others were obtained from the Arabidopsis Biological Resource Center. All seeds were stored in our laboratory.
Novel plant genotypes	BnaA07.MKK9DD-OE, BnaC03.MPK3-OE, BnaC03.MPK6-OE, Bnamkk9 knock out mutant, BnaC03.MPK3-OE/Bnamkk9 and BnaC03.MPK6-OE/Bnamkk9 were generated by Agrobacterium-mediated hypocotyl method as described in manuscript. The Csv::BnaA07.MKK9DD transgenic Arabidopsis plants were generated by A. tumefaciens-mediated floral dipping as described in manuscript. The Csv::BnaA07.MKK9DD/AtMPK3SR were generated by crossing.
Authentication	The transcript abundance of the OE lines was detected by RT-PCR or RT–qPCR. The T-DNA insertion mutant was validated with T-DNA border primers and gene-specific primers. The Bnamkk9 KO mutants were identified by PCR and Sanger sequencing.
